# Back pain symptom relieved by tubular lumbar microdiscectomy

**DOI:** 10.3389/fsurg.2025.1702002

**Published:** 2026-01-27

**Authors:** Yan-Wei Jiang, Mao-chao Zhou

**Affiliations:** Department of Neurosurgery, Fujian Medical University Union Hospital, Fuzhou, Fujian, China

**Keywords:** low back pain, lumbar disc herniation, modic change, tubular discectomy, Visual Analogue Scale (VAS)

## Abstract

**Background:**

This study aimed to determine the impact of discectomy on back pain and to identify the factors associated with back pain improvement.

**Methods:**

A retrospective analysis was conducted on patients with lumbar disc herniation who underwent lumbar microdiscectomy at Fujian Medical University Union Hospital. Visual Analogue Scale (VAS) scores for back and leg pain were assessed before and after the tubular lumbar microdiscectomy.

**Results:**

A total of 111 patients were included in this study. Pre- and post-operative VAS scores for back pain were 4.86 and 2.59, respectively. For radicular leg pain, pre- and post-operative VAS scores were 4.86 and 2.59, respectively. Univariate and multivariate analyses showed that the preoperative lumbar VAS score was significantly associated with improvement in back pain after tubular discectomy.

**Conclusion:**

Tubular microdiscectomy significantly alleviated back pain symptoms in patients with lumbar disc herniation. The findings of this study may help spine surgeons in better educating patients regarding post-operative expectations following this surgical procedure.

## 1 Introduction

Back and radicular pain are common symptoms of lumbar disc herniation (LDH) (1, 2). Nerve compression can cause neurological symptoms, including pain and numbness. Lumbar microdiscectomy, the gold standard procedure for the treatment of LDH, relieves nerve compression while preserving spinal stability (3). However, the impact of discectomy on low back pain (LBP) secondary to LDH remains unclear.

Spine surgeons generally recommend spinal fusion for patients with LDH presenting with back pain (4, 5). Conversely, some surgeons argue that decompression combined with discectomy is sufficient for managing these patients (6–8). The prognosis of lumbar microdiscectomy has not yet been determined. Surgeons aim to identify patient subgroups who may benefit from tubular microdiscectomy without the need for spinal fusion (9).

A precise understanding of the relationship between lumbar microdiscectomy and LBP alleviation is essential for managing patient expectations. This investigation aimed to characterize the postoperative course of LBP following discectomy, determine modifiable factors influencing back pain recovery, and provide surgeons and patients with actionable insights into procedural outcomes.

## 2 Methods

### 2.1 Study design

The study protocol was approved by the Fujian Medical University Union Hospital ethics committee (approval number: 2023WSJK007). This retrospective study included patients with LDH who underwent lumbar microdiscectomy at our center. Patients with a prior lumbar surgery history were excluded from the study, and all eligible candidates underwent routine preoperative dynamic spinal radiography to rule out spinal instability before surgical intervention. Patient characteristics, including sex, age, smoking, alcohol consumption, operation segment, operation time, and hospitalization duration, were recorded. Both LBP and leg pain were measured using the Visual Analogue Scale (VAS), a widely used instrument for assessing pain intensity (8). The VAS allows patients to express their pain levels on a continuous scale. VAS assessments were performed preoperatively and 1 year after surgery. Modic changes were re-evaluated based on preoperative findings on magnetic resonance imaging (MRI) (10). The Michigan State University (MSU) grade was re-evaluated using preoperative MR findings. This assessment is crucial, as it provides a more accurate classification of LDH, taking into account both the size and medial-to-lateral location of the herniation on axial images (1).

### 2.2 Surgical procedure

C-arm fluoroscopy was used to locate the surgical segment and puncture site. Following progressive dilatation, a working channel was established. The surgeon performed a lumbar microdiscectomy using a microscope to visualize the surgical field. Following identification of the anatomical landmarks, the soft tissues were carefully dissected to expose the lamina. Once the bony structure was successfully removed, the ligamentum flavum was excised using a lamina rongeur, exposing the dural sac and nerve root. After identifying the nerve root, the herniated disc was removed. The nerve root and dura mater were pulsated sufficiently after decompression.

### 2.3 Statistical analyses

All statistical analyses were performed using R software (version 4.0.5; R Foundation for Statistical Computing, Vienna, Austria). Multivariate and univariate analyses were performed to determine covariates. The *χ*^2^ test and t-test were used for bivariate analyses of categorical and continuous variables, respectively. Figures were generated using GraphPad Prism 8.0.2 (GraphPad Software, Inc., San Diego, CA, USA). Sample characteristics are presented as numbers and percentages. Statistical significance was set at *p* < 0.05.

## 3 Results

Patient characteristics are presented in Table 1. A total of 111 patients were included in this study, comprising 52 (46.8%) women. Twenty patients (18%) were smokers, and five (4.8%) reported alcohol consumption. Sex, age, body mass index (BMI), smoking, and Modic change were not associated with symptom improvement (Table 2). The lumbar VAS score was significantly associated with improvement in back pain after surgery (*p* = 0.021). In addition, a history of alcohol abuse was significantly associated with symptom improvement (*p* = 0.022). The most common levels of LDH were L4/L5 (58.6%) and L5/S1 (33.3%).

**Table 1:** Patient characteristics.

Variable	*N*	%
Gender		
Men	59	53.2
Women	52	46.8
Alcohol	5	4.5
Smoke	20	18.0
Level		
L1/L2	1	0.9
L2/L3	1	0.9
L3/L4	7	6.3
L4/L5	65	58.6
L5/S1	37	33.3

**Table 2:** Univariate analysis and multivariate analysis of risk variables.

Variable	Univariate analysis *p*-value	Multivariate analysis	
		OR (95% CI)	*p*-value
Male	0.355	0.666 (.238–1.868)	0.440
Age	0.150	0.997 (0.963–1.061)	0.849
BMI	0.583	1.072 (0.902–1.273)	0.431
Diabetes	0.338	0.506 (0.126–1.989)	0.329
Alcohol	0.022	0.027 (0.001–0.588)	0.022
Smoke	0.980	6.215 (0.691–55.913)	0.103
Lumbar VAS	0.012	2.166 (1.124–4.174)	0.021
Modic change	0.110	0.638 (0.209–1.953)	0.432

**Figure 1:**
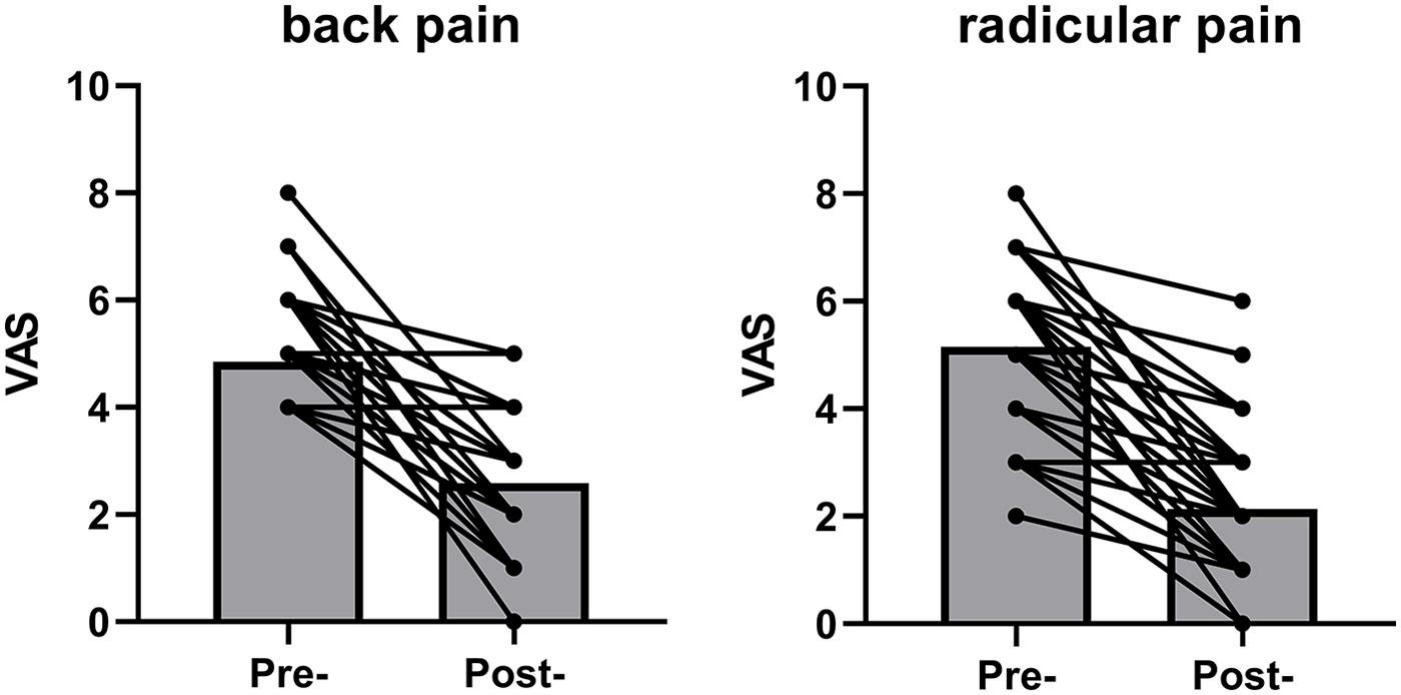
Improvement in low back pain severity pre- and post-operatively.

**Figure 2:**
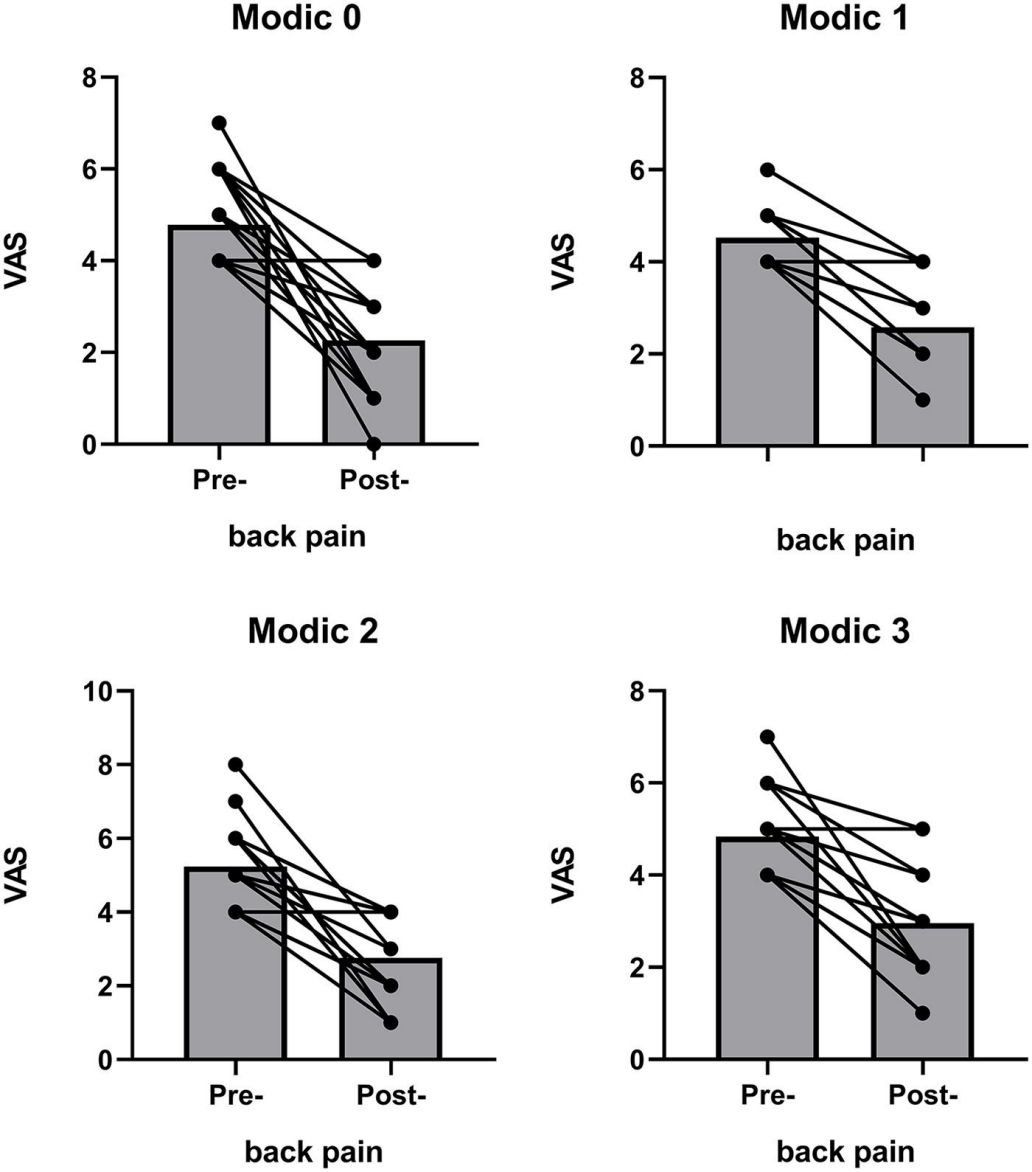
Improvement in low back pain severity pre- and post-operatively by subgroup.

**Figure 3:**
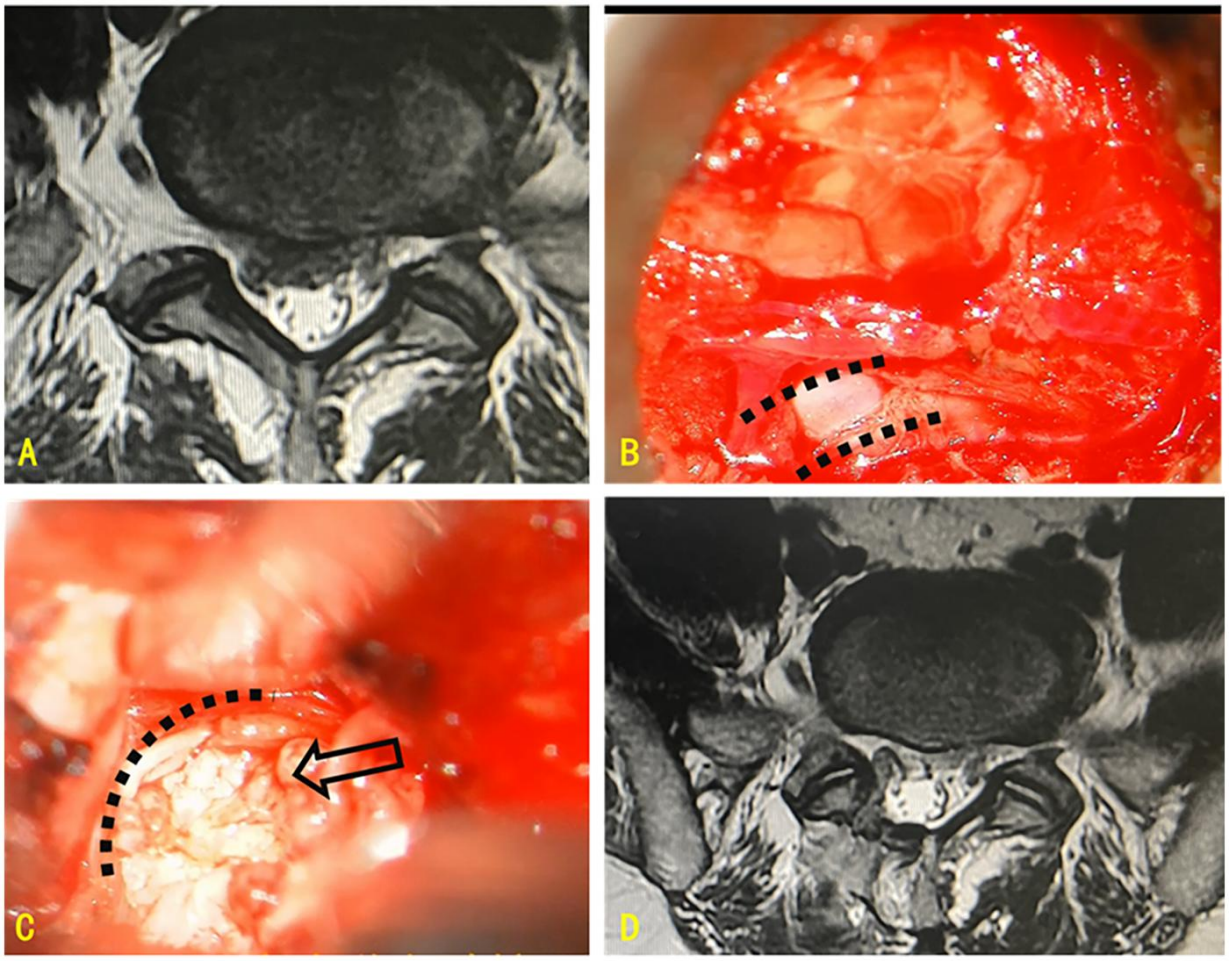
**(A)** lumbar disc herniation compressing the right nerve root sleeve; **(B)** microscopic image showing exposure of the right nerve root sleeve (dashed line); **(C)** retraction of the nerve root sleeve (dashed line) for resection of the herniated disc (arrow); **(D)** postoperative imaging demonstrating resection of the herniated disc and resolution of nerve root compression.

Both back and leg pain VAS scores improved after lumbar microdiscectomy (Figure 1). Improvement in the back pain VAS score was also observed in the subgroup of participants with Modic changes (Figure 2). Figure 3 shows preoperative and intraoperative images of the herniated intervertebral disc and its surgical resection. The mean VAS score for back pain was 4.86 preoperatively and 2.59 postoperatively (Table 3). The mean VAS for radicular leg pain improved from 4.86 preoperatively to 2.59 after lumbar microdiscectomy (Table 4). In the MSU subgroups, both back and leg pain improved.

**Table 3:** Comparison of pre- and postoperative low back pain severity.

Group Category	Pre-operative	Post-operative	P
Total	4.86	2.59	<0.05
Modic Change	4.90	2.78	<0.05
Without Modic Change	4.79	2.27	<0.05
MSU grade 1	4.64	2.81	<0.05
MSU grade 2	4.87	2.47	<0.05
MSU grade 3	5.04	2.40	<0.05

**Table 4:** Comparison of pre- and postoperative radicular leg pain severity.

Group Category	Pre-operative	Post-operative	*P*
Total	5.15	2.14	<0.05
Modic Change	5.10	2.188406	<0.05
Without Modic Change	5.24	5.24	<0.05
MSU grade 1	5.17	2.22	<0.05
MSU grade 2	5.27	2.02	<0.05
MSU grade 3	4.92	2.18	<0.05

## 4 Discussion

Leg pain and LBP are the two most common symptoms of LDH (7, 11, 12). The primary purpose of lumbar microdiscectomy is to decompress the nerves (9). In this study, we demonstrated that tubular discectomy significantly relieved low back symptoms. We also found that a history of alcohol abuse and preoperative back pain severity were the only two factors associated with postoperative pain relief.

Many factors contribute to LBP (13), including discs, endplates, facet joints, and muscles. In the present study, patients with lumbar instability were excluded. For those with partial Modic changes or facet joint capsule lesions, the same surgical strategy was adopted. We observed that these patients still achieved partial relief from LBP symptoms following the tubular lumbar surgery. The advantage of tubular microdiscectomy over open surgery is its ability to significantly reduce intraoperative bleeding and minimize disruption to surrounding tissue structures (9). The minimally invasive procedure may reduce the risk of postoperative iatrogenic lumbar spondylolisthesis. Unlike the previous studies, the present study demonstrated that tubular discectomy was beneficial for all types of Modic changes.

In this study, preoperative back pain severity was identified as a predictor of symptom improvement. Patients with more severe symptoms were more likely to benefit from surgery. Inflammation plays a significant role in the pathology of LDH (14). Similar to other decompression surgeries, tubular microdiscectomy may reduce local canal and nerve inflammation (15–17). Inflammation plays a crucial role in the mechanisms of pain and spinal degeneration. The local release mechanisms of inflammatory mediators in the lumbar spine are complex and remain an active area of research. Future studies should clarify the role of cytokines in the inflammatory process and their relationship with pain.

In our study, increased alcohol consumption was associated with worse outcomes. This may be due to changes in venous flow, as enlargement of the epidural venous plexus is commonly observed during tubular discectomy (18). Individuals with a history of alcohol abuse may experience impaired venous flow compared to those without such a history.

Our study has several limitations. First, due to the retrospective design, the study is subject to inherent biases. Second, the sample size was relatively small, particularly within each Modic-grade subgroup, which limited the ability to perform a valid statistical analysis. For the included 111 patients, the statistical power for odds ratio (OR) testing is 75%, which is close to the target power of 80% and therefore acceptable for the primary study objectives. Finally, the diversity and representativeness of the study population may affect the generalizability of the results, which should be further addressed in future studies.

## 5 Conclusion

A simple tubular microdiscectomy may relieve back pain in patients with lumbar disc disease. This study reveals patient outcomes following microdiscectomy. The findings of this study may help spine surgeons educate patients more effectively regarding postoperative expectations associated with this surgical procedure.

## Data Availability

The raw data supporting the conclusions of this article will be made available by the authors, without undue reservation.
